# Modulations in Oscillatory Activity of Globus Pallidus Internus Neurons During a Directed Hand Movement Task—A Primary Mechanism for Motor Planning

**DOI:** 10.3389/fnsys.2019.00015

**Published:** 2019-04-30

**Authors:** Shreya Saxena, Sridevi V. Sarma, Shaun R. Patel, Sabato Santaniello, Emad N. Eskandar, John T. Gale

**Affiliations:** ^1^Zuckerman Mind Brain Behavior Institute, Columbia University, New York, NY, United States; ^2^Neuromedical Control Systems Laboratory, Department of Biomedical Engineering, Institute for Computational Medicine, Johns Hopkins University, Baltimore, MD, United States; ^3^Genetics and Aging Research Unit, Department of Neurology, McCance Center for Brain Health, Harvard Medical School, Massachusetts General Hospital, Boston, MA, United States; ^4^Biomedical Engineering Department, CT Institute for the Brain and Cognitive Sciences, University of Connecticut, Storrs, CT, United States; ^5^Leo M. Davidoff Department of Neurological Surgery, Albert Einstein College of Medicine, The Bronx, NY, United States; ^6^Department of Neurosurgery, Emory University, Atlanta, GA, United States

**Keywords:** basal ganglia, globus pallidus internus (GPi), beta-band, motor control, movement planning

## Abstract

Globus pallidus internus (GPi) neurons in the basal ganglia are traditionally thought to play a significant role in the promotion and suppression of movement via a change in firing rates. Here, we hypothesize that a primary mechanism of movement control by GPi neurons is through specific modulations in their oscillatory patterns. We analyzed neuronal spiking activity of 83 GPi neurons recorded from two healthy nonhuman primates executing a radial center-out motor task. We found that, in directionally tuned neurons, the power in the gamma band is significantly (*p* < 0.05) greater than that in the beta band (a “cross-over” effect), during the planning stages of movements in their preferred direction. This cross-over effect is not observed in the non-directionally tuned neurons. These data suggest that, during movement planning, information encoding by GPi neurons may be governed by a sudden emergence and suppression of oscillatory activities, rather than simply by a change in average firing rates.

## Introduction

A central question in motor neuroscience is how the best action is selected at any given moment while carrying out a voluntary movement. The basal ganglia (BG) neurons are thought to play a significant role in movement selection, wherein the globus pallidus internus (GPi) neurons form a major structure (Kandel et al., 2000). A widely accepted theory suggests that for any given state, there is a range of possible and competing actions, and the BG participate in the process of selecting the most desirable or profitable action given the current context and prior learning in a “center-surround” model (Nambu, 2004). Specifically, the theory suggests that modulations in the firing rate of task-related GPi/substantia niagra neurons signal the promotion of desired movements and the suppression of unwanted movements (Albin et al., 1989; DeLong, 1990; Mink, 1996; Nambu, 2004). In this study, however, we hypothesize that movement control occurs via modulations in *oscillatory activity* in the BG neurons, more specifically in the “beta” (15–30 Hz) and “gamma” (35–90 Hz) bands.

Several experiments have demonstrated the modulation of GPi neurons' firing rates to direction-specific movements as well as reward information (Bromberg-Martin et al., 2010; Shin and Sommer, 2010; Tachibana and Hikosaka, 2012; Howell et al., 2016). Moreover, numerous studies have focused on the potential role of beta oscillatory activity in the basal ganglia, in both single unit and local field activities, in the pathophysiology of PD (Miller and DeLong, 1987; Filion and Tremblay, 1991; Levy et al., 2000, 2002; Brown et al., 2001). However, few studies have examined the role of beta oscillatory activity of basal ganglia in normal function (Courtemanche et al., 2003; Feingold et al., 2015). To this end, we examined single-unit activity of 83 GPi neurons in two naive non-human primates engaged in a radial center-out motor task. We set out to ascertain the functional relationship between movement and oscillatory activity in beta and gamma bands in the healthy condition. Here, we refer to “oscillatory activity” as modulations in the power spectrum of individually recorded neurons in a specific frequency band.

In the directionally tuned, i.e., task-related neurons, our results show a significant increase in gamma power as compared to beta power (*p* < 0.05), specifically during the planning of movement. This trend is not observed in the non-directionally tuned neurons. This suggests that the GPi neurons involved in the planning of movement communicate information through a “cross-over” effect, i.e., an emergence in gamma oscillatory activity with a concurrent suppression in beta oscillatory activity. A cross-over effect has previously been observed in other parts of the motor circuit, specifically the motor and premotor cortex (Schoffelen et al., 2005; Donner et al., 2009), as well as in a preliminary analysis of a subset of the data used in the present study (Saxena et al., 2011).

The data in this study are the first to demonstrate that beta and gamma modulation in the GPi is direction specific and that movements are encoded in the temporal domain at the level of single-unit activities. Hence, the interaction between beta and gamma oscillatory activity may serve to encode additional orders of information, not encoded in the firing rate domain, as originally hypothesized.

## Materials and Methods

### Experimental Methods

Two healthy adult male Rhesus monkeys (*macaca mulatta*) were trained to perform a radial “center-out” motor task; more details below. “Center-out” tasks were originally developed by (Georgopoulos et al., 1988), but have used extensively in subsequent studies (Georgopoulos et al., 1988; Truccolo et al., 2008). Both animals were independently housed in a climate and light controlled environment. Target structures were localized using magnetic resonance imaging (MRI) and recording chambers were placed stereotactically, under isoflourine using sterile technique, such that the electrode trajectory avoided sinus and ventricle space. One chamber was centered at A13, L15, aligned vertically to allow a dorsal approach to the GP. The other chamber was centered at A11, with an approach of approximately 40 degrees relative to vertical (roughly normal to the skull). An MRI image (MPRAGE; TR 11.1; TE 4.3/1; TA 13:37) was obtained after the recording chamber had been implanted, using mineral-oil filled capillary tubes placed at known grid positions as fiducial markers. For the angled chamber, penetrations were advanced until the GP was encountered. For the vertical chamber, separate penetrations were made medial to the putaminal sites to positively identify the GP. GP units were clearly identified from putaminal units by their much higher spontaneous firing rates (DeLong, 1971). In addition to the chamber placement, scleral search coil (Judge et al., 1980) were implanted to allow for accurate measure of eye position (Crist Instruments, Bethesda MD). All animal procedures were performed in accordance with the National Institutes of Health guidelines and the Institutional Animal Care and Use Committee approved study protocol.

Each day, the animals sat in a chair that was placed into a sound-attenuating enclosure facing an LCD computer monitor. A sipper-tube was positioned at the tip of their mouth for reward delivery and a joystick was fixed to the chair for the animal to manipulate during the task. In addition, a shielding plate was positioned next to the joystick such that the animal was only able to manipulate the joystick using the hand contralateral to the recording chamber. The animals were then trained 5–6 days a week on a visual-motor task.

**Figure 1:**
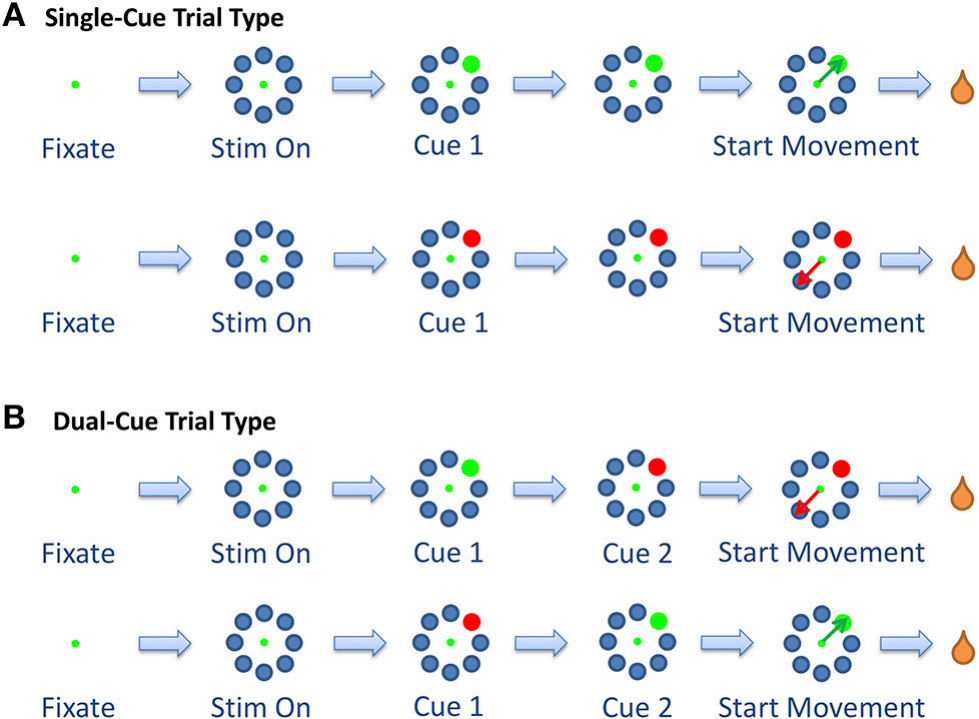
The two trial types. **(A)** The single-cue trial type consists of trials in which the stimulus did not change throughout the trial. **(B)** The dual-cue trial type consists of the trials in which the stimulus changed colors. Figure adapted from Saxena et al. (2011).

To initiate a trial, the animals were required to fixate (“F”) and position the cursor on a central fixation point for a period of 200–300 ms. At this point, eight gray objects appeared in a radial arrangement equidistant from the center of the screen, signifying stimulus on (“S”). While maintaining gaze on the fixation point for a period of another 200–300 ms, a random gray target was replaced by a stimulus cue (“Cue 1”). The stimulus cue was either a green or red circle, which instructed the animal to choose that target (for the green circle) or the diametrically opposing target (for the red circle). As an additional setting, dual-cued trials were also added to the task. In these trials, the first cue would be replaced by a second cue (“Cue 2”) that instructed the animal to change the target selection. The second cue would appear 100–900 ms after the first cue. Trials with only one cue are denoted as *single-cue* trials and trials with two cues are denoted as *dual-cue* trials (Figure 1).

The start of movement is denoted by “M.” The trial was concluded when the primate selected a target. In the *dual-cue* trial type, the trial was concluded without a liquid reward if the primate started movement before the target light changed in color. If at any point the animal broke fixation, prematurely moved the joystick or failed to select the correct target (in the required time), the trial was aborted and the animal was not rewarded. The animals were water-deprived, and the trials that were completed correctly were followed by a liquid reward (water). The animal's arm was not immobilized while moving the joystick. Target positions and movement types were randomized such that many movements toward each of the eight positions could be analyzed over the course of a single recording session. An average of 53% (55%) of the successful trials in any session were single-cue trials for Monkey 1 (Monkey 2).

Once the animal had been fully trained on the behavioral task, extracellular microelectrode recordings were made from the GPi while the primates performed the behavioral task. Electrodes (300–500 KOhm metal micro-electrodes; FHC, Bowden, ME) were introduced into the brain through a 1 mm spaced grid (Crist Instruments, Bethesda, MD). Neurons were not preselected for task-specific modulation, assuring random sampling of GPi neurons. Instead, the electrode was advanced until the activity of one or more neurons was well-isolated. The localization of the GPi was based both on MRI positioning information (as detailed above) and neurophysiological characteristic, such as high irregular firing rate and lack of pause-burst spiking patterns (which are characteristic of globus pallidus externus). In any given session, the activity of up to three neurons was recorded from a single electrode. Single electrode recordings were repeated on a semi-daily basis for the duration of the study.

Neurophysiological activity was digitized and high-pass filtered at 0.2–6.5 KHz through the head-stage and continuously stored, along with behavioral events, by a PowerLinc 1401 acquisition system (Cambridge Electronic Design, Cambridge UK) at 20 kHz. Offline, the continuous data was parsed into single neuron records using an offline sorting algorithm (Spike2, Cambridge Electronic Design, Cambridge UK). To do this, data was thresholded to identify spike events from noise and clustered using the first and second principal components of the waveform signal. Data was disregarded if the recording was unstable or if individual single unit activity was indiscernible from noise or multi-unit activity.

### Data Analysis

We considered the two trial types, *single-cue* and *dual-cue*, separately. We first built point process models (PPMs) for the activity of each neuron as the primate was reaching in the 8 directions. We then used specific parameters of these PPMs to determine directional tuning of the neuron for each trial type. Finally, we computed the population-averaged power in the beta and the gamma frequency bands in overlapping windows throughout the entire trial for both directionally tuned neurons and the non-directionally tuned neurons. The details are provided in the following sections.

### Determining Directional Tuning

Point process methods have been used to analyze the spike train activity for a broad range of neural systems (Sarma et al., 2010, 2012; Saxena et al., 2010, 2012; Santaniello et al., 2012; Sumsky et al., 2017; Sumsky and Santaniello, 2018). A neural spike train can be treated as a stochastic series of random binary events (i.e., the spike times) continuously occurring in time, otherwise known as a point process (Truccolo et al., 2005; Coleman and Sarma, 2010; Sarma et al., 2010).

The spike train can be discretized into bins of length Δ, and if Δ is small enough, we are left with a discrete time series of 1 and 0 s. In this case, the 1 s are individual spike times and the 0 s are the times at which no spikes occur. To define a point process model (PPM) of neural spiking activity, an observation interval (0,T] is considered to be the length of the spike train, and N(t) is allotted to be the number of spikes counted in interval (0,t] for t ∈ (0,T]. A PPM of a neural spike train is completely characterized on a given observation interval (0,T] by defining the conditional intensity function (CIF) (Snyder and Miller, 2012). The timings between spike events can be described as a stochastic point process and its probability distribution is characterized by a rate function, λ(t|·), formally known as the CIF, defined as:

$$\begin{array}{l} \lambda \left(\text{t}|{\text{H}}_{t}\right)≜\underset{\;\Delta \;\to 0}{\lim}\frac{\text{Pr}\left(N\left(t+\;\Delta \;\right)-N\left(t\right)=1|{\text{H}}_{t}\right)}{\;\Delta \;}, \end{array}$$

where H_*t*_ is a vector comprising the relevant covariates in the past and up to including time *t*, and Pr the probability. PPMs have been extensively used to extract temporal patterns and non-stationarities in spiking data (Sarma et al., 2010, 2012; Saxena et al., 2011, 2012; Santaniello et al., 2012). In these studies, the CIF is modeled as an explicit function of extrinsic and intrinsic factors, and can be estimated directly, via maximum likelihood estimation (Truccolo et al., 2005; Coleman and Sarma, 2010; Sarma et al., 2010) from extracellular *in-vivo* recordings. Estimating λ (t|H_t_) is equivalent to estimating of the entire probability distribution of the spiking activity, and is thus more powerful than the traditional calculations of first- and second- order statistics of the spike train.

In this study, we calculated the probability of spiking of each neuron as a function of the stimulus information and the neuron's own spiking history. Specifically, at each time window, the CIF was expressed as

$$\begin{array}{l} \lambda \left(t|H_{t},\Theta \right)=\lambda^{s}\left(t|\Theta \right)\cdot \lambda^{H}\left(t|H_{t},\Theta \right) \end{array}$$

Where λ^*S*^(t| Θ) describes the effect of the movement direction stimulus on the neural response and $\lambda^{H}\left(\text{t}|{\text{H}}_{t},\;\Theta \;\right)$ describes the effect of spiking history on the neural response. Θ is a parameter vector to be estimated from data, using maximum likelihood methods.

The following structure for λ^*S*^ was used to model the history-independent component, i.e., the stimulus component, in each window.

$$\begin{array}{l} \log\lambda^{S}\left(\text{t}|\;\alpha \;,\text{d}\right)=\alpha_{d},d\in 1,\ldots ,8\;, \end{array}$$

where movement direction d = 1, 2, …8, corresponds to 0, 45, 90, …315 degrees clockwise from the “Up” direction, respectively, and α_*d*_ is a scalar.

The history-dependent component was modeled in the following manner in each time window.

$$\begin{array}{l} \log\lambda^{H}\left(\text{t}|\;\varphi \;,\;\gamma \;,\;\beta \;\right)=\overset{9}{\underset{j=0}{\sum }}\varphi_{j}n\left(t-j:t-\left(j+1\right)\right)\\ +\overset{8}{\underset{k=0}{\sum }}\gamma_{k}n\left(t-\left(2k+12\right):t-\left(2k+14\right)\right)\\ +\overset{8}{\underset{l=0}{\sum }}\beta_{l}n\left(t-\left(5l+30\right):t-\left(5l+35\right)\right), \end{array}$$

where *n*(*a*:*b*) is the number of spikes observed in the time interval [*a, b*) during the epoch. The ${\left\{\varphi_{j}\right\}}_{j=0}^{9}$ parameters measure the effects of spiking history in the previous 10 ms and therefore can capture refractoriness and / or bursting on the spiking probability in the given time window (Sarma et al., 2010, 2012; Santaniello et al., 2012). The ${\left\{\gamma_{k}\right\}}_{k=0}^{8}$and the ${\left\{\beta_{l}\right\}}_{l=0}^{8}$ parameters capture longer-term history effects such as oscillatory activity between 10 and 100 Hz. We estimated the following parameter vector using maximum likelihood methods.

$$\begin{array}{l} \;\Theta \;=\left[{\left\{\alpha_{d}\right\}}_{d=1}^{8},{\left\{\varphi_{j}\right\}}_{j=0}^{9},{\left\{\gamma_{k}\right\}}_{k=0}^{8},{\left\{\beta_{l}\right\}}_{l=0}^{8}\right] \end{array}$$

Each PPM was estimated during 80% of the trials, and the goodness-of-fit was assessed on the remaining 20% of the trials (cross-validation) with the Kolmogorov Smirnov (KS) plot after time rescaling of the spike trains (Brown et al., 2002). Only neurons with PPMs whose KS plots were within the 95% confidence bounds were included in this study; the summary statistics are provided in Table 1. For more details on fitting PPMs, see (Brown et al., 2002; Sarma et al., 2010, 2012; Santaniello et al., 2012).

**Table 1:** Time taken for movement planning and completion of movement for Monkey 1.

**Category**	**Single-cue trials**	**Dual-cue trials**
Median time taken from last cue to start of movement (ms)	839 (894)	537 (505)
Median time taken from start of movement to reward (ms)	299 (423)	289 (413)

*Values in parentheses for Monkey 2*.

To establish direction tuning, we inquired whether, given the same spiking history, the spiking activity of a neuron was significantly different when the primate was moving in one of the eight target directions. If the history-independent parameter in one direction was found to be significantly different from at least four other directions at a 95% confidence level, the neuron was determined directionally tuned. Thus, we examined the history-independent parameters α_*d*_. rresponding to each direction of movement of the primate.

Specifically, for each direction *d*′ = 1, …, 8, $p_{d^{\prime }d}=\mathrm{Pr}\left(e^{\alpha_{d^{\prime }}}>e^{\alpha_{d}}\right)=\mathrm{Pr}\left(\alpha_{d^{\prime }}>\alpha_{d}\right)$ was computed for *d* ≠ *d*′. $p_{d^{\prime }d^{\prime }}$ was defined as 0. The Gaussian approximation was used, which is one of the asymptotic properties of maximum likelihood estimates to compute $p_{d^{\prime }d}$ (Brown et al., 2003). Let *N*_*d*_ be the number of *d*′ ∈ 1, …, 8 for which $p_{d^{\prime }d}>0.975$. If *N*_*d*_ ≥ 4 for any *d* ∈ 1, …, 8, then the neuron was determined directionally tuned in this direction, now termed d^*^. If more than one direction was tuned in the neuron, then the following formula was used.

$$\begin{array}{l} d^{*}=\underset{d}{\text{argmax}}|\alpha_{d}-\frac{\sum_{i=1}^{8}\alpha_{i}}{8}|,d\in 1,\ldots ,8 \end{array}$$

We first computed the percentage of directionally tuned neurons in each time window studied for each of the four movement types presented in the task (cf. Figure 1). We identified the epoch e^*^ for which each movement type had the maximum percentage of directionally tuned neurons (Supplementary Figure 1). In the subsequent analysis, a neuron was classified as directionally tuned in a trial type (i.e., *single-cue* or *dual-cue*) if it was directionally tuned for either movement type in this epoch e^*^. Only the neurons which were recorded during both movement types in a trial type were kept in the analysis.

### Determining the Presence of a Cross-Over Effect Using Traditional Spectral Analysis

The analyses were performed separately for directionally tuned neurons and for non-directionally tuned neurons, for each trial type: *single-cue* and *dual-cue*. Oscillatory characteristics of the neurons in the beta (15–30 Hz) and gamma (35–90 Hz) frequency band were assessed by using the power spectrum density (PSD) with the Welch method (Welch, 1967). Given a neuron and a task type (i.e., *single-cue* or *dual-cue* tasks), for each task-related marker *m* (*single-cue* tasks: *m* = {*F, S, Cue*_1_, *M*}; *dual-cue* tasks: *m* = {*F, S, Cue*_1_, *Cue*_2_, *M*}, Figure 1), the spike trains around *m* were divided into 9 overlapping segments (length: 512 ms; step size: 50 ms) centered from *m*−144 to *m*+256 ms and each segment was multiplied by a Hanning window of equal length. The PSD of the neuron in each time window was computed as the average periodogram across the number of trains available in that window (Welch, 1967). Finally, the signal-to-noise ratio (SNR) in that window was computed at each frequency *f* according to the formula (Gale et al., 2009):

(1) $$\begin{array}{r} SNR\left(f\right)≜\frac{PSD\left(f\right)-\mu }{\sigma }, \end{array}$$

where *PSD*(*f*) is the PSD at frequency *f* and μ (σ) is the mean (standard deviation) of the PSD across all the frequencies. Only frequencies for which SNR is ≥1.5 were considered significant and included in this study. For each time window, the average power of the neuron in the beta (gamma) frequency band was estimated as the average value of *SNR*(*f*) in the interval [15, 30] Hz ([35, 90] Hz). If the neuron was directionally tuned, only trains recorded during tasks involving the tuned direction were considered, otherwise all the recorded spike trains of the neuron were considered.

#### Determining the Presence of a Cross-Over Effect Using PPMs

For the analysis of prominent oscillatory activity, different PPMs were constructed for directionally tuned and non-directionally tuned neurons.

If the neuron was determined to be directionally tuned, a *Direction-Specific* model was constructed for that neuron using *only* the data from the trials where the primate was reaching in direction d^*^, where d^*^ is the tuned direction as determined above. The model structure for the history-independent term was defined as logλ^*S*^(t| α) = α. The model structure for the history-dependent term λ^*H*^ remained the same as above.

If the neuron was not tuned in any direction, model structures remained the same as above, and the *Multi-Direction* PPM was used to determine the presence of oscillatory activity.

We first determined the presence of beta and gamma oscillatory activity for each neuron. The parameters ${\left\{\gamma_{k}\right\}}_{k=0}^{8}$, corresponding to the history bins −12 to −30 ms from right to left measure the effects of spiking history in the previous 12–30 ms, and therefore can capture the presence of oscillatory activity in the frequency range of 33–83 Hz. This corresponds to the gamma frequency band, and the presence of gamma oscillatory activity was determined if any one of the parameters representing oscillatory activity in this frequency range was significantly higher than 1, that is, for at least one $k\in 0,\ldots 8,LB_{k}^{\gamma }>1$, $LB_{k}^{\gamma }\leq e^{\gamma_{k}}$. $LB_{k}^{\gamma }$ is the 95% lower confidence bound for parameter γ_*k*_.

Similarly, we analyzed parameters ${\left\{\beta_{l}\right\}}_{l=0}^{8}$, capturing recurrent patterns with period −30 to −75 ms, corresponding to the beta frequency band. The presence of beta oscillatory activity was determined if any one of the parameters representing oscillatory activity in this frequency range was significantly higher than 1, that is, for at least one $l\in 0,\ldots 8,LB_{l}^{\beta }>1$, $LB_{l}^{\beta }\leq e^{\beta_{l}}$. $LB_{l}^{\beta }$ is the 95% lower confidence bound for parameter β_*l*_.

Next, we determined whether the neuron has a higher tendency to display gamma oscillatory activity or beta oscillatory activity. If a neuron has no parameters significantly higher than 1 in the beta band, but does in the gamma band, it automatically has a higher tendency to display gamma oscillatory activity than beta. However, if a neuron had significant parameters in both bands, we compared the lower bounds of the highest parameters in both bands to determine whether it had a higher tendency to oscillate in the beta band or the gamma band, i.e., if $\underset{k}{\max}\left(LB_{k}^{\gamma }\right)>\underset{l}{\max}\left(LB_{l}^{\beta }\right)$ for *k, l* ∈ 0, …8, then the neuron has a higher tendency to oscillate in the gamma band.

Finally, we separately calculated the percentage of directionally tuned and non-directionally tuned neurons that had a higher tendency to have gamma oscillatory activity than beta oscillatory activity. We calculated this percentage for each overlapping window as described in the previous section. Thus, we could infer the suppression and increase of oscillatory activity in the gamma and beta bands across the trial. For comparison, we also calculated the percentage of directionally tuned and non-directionally tuned neurons in each time window that had a higher tendency to have beta oscillatory activity than gamma oscillatory activity.

The same statistic was also computed for randomized spike trains, built by randomly shuffling the inter spike intervals of the original spike trains for each trial of each neuron a total of 100 times. We calculated the 5 and 95% bounds of the percentage of neurons falling in each bin from these randomized spike trains.

## Results

A total of 83 neurons were isolated from the GPi from two non-human primates (Monkey 1, *n* = 27; Monkey 2, *n* = 56) as the animals performed the task. Monkey 1 (2) performed a total of 18,978 (28,303) trials, out of which 14,370 (21,183) were successful across both trial types over 31 (50) days. Monkey 1 (2) had an average success rate of 77 ± 7% (75.2 ± 6%) trials per recorded day. On average each animal performed 16.39 ±6.53 successful trials per day, per direction of movement. These successful trials are analyzed in this study. Some further behavioral statistics for each animal are provided in Table 1.

**Figure 2:**
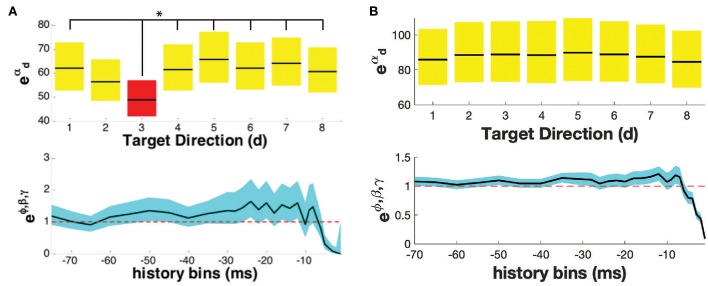
Point process model for **(A)** a directionally tuned neuron, **(B)** a non-directionally tuned neuron. Both **(A,B)** show the propensity of the neuron to fire at time t, given the movement direction (top row), and the neuron's own history (bottom row). The parameters of the PPM are given in black, while the 95% confidence bounds are given in yellow/ red/ blue. In **(A)**, the neuron is tuned in the direction with an asterisk, highlighted in red, i.e., direction d_3_. In **(B)**, the neuron is not tuned in any direction.

For each neuron, the directional tuning for each trial type was calculated. We identified the epoch e^*^ for which each movement type had the maximum percentage of directionally tuned neurons (Supplementary Figure 1). Figure 2 shows the PPM of two example neurons in one trial type. In the top row of Figures 2A,B, parameters $e^{\alpha_{d}},d=1,\ldots 8$, account for the history-independent, non-oscillatory component of the discharge rate of a single neuron when the primate is reaching in direction *d*. In Figure 2A, the history-independent parameter in direction *d*_3_ is significantly different from those in 6 other directions. Thus, this neuron is determined tuned in the direction *d*_3_. Note that directionally tuned neurons may have a significantly *lower* or *higher* parameter $e^{\alpha_{d}}$. In Figure 2B, none of the history-independent parameters are significantly different from those in any other direction; this neuron is thus not directionally tuned. Supplementary Figure 2 shows the percentage of neurons displaying tuning in each direction. Supplementary Figure 3 shows an example raster plot of a directionally tuned neuron, where we also see an inhibition in the firing activity of the neuron in the tuned direction within the relevant epoch.

Table 2 shows the number of neurons that fell in each category. We accepted a neuron as significantly tuned for a trial type if it demonstrated directional tuning during either movement cue.

**Table 2:** Number of neurons displaying directional tuning in one or both movement cues (i.e., while the subject is moving toward the green target or away from the red target), for each trial type.

**Category**	**Single-cue trials**	**Dual-cue trials**
Directionally tuned for both movement cues	35 (47)	31 (37)
Directionally tuned for only one movement cue	26 (31)	24 (29)
Non Directionally tuned for both movement cues	19 (23)	25 (30)
Not enough data to successfully cross-validate PPM	3 (4)	3 (4)
Total	83 (100)	83 (100)

*Values in parenthesis represent percent of neurons*.

**Figure 3:**
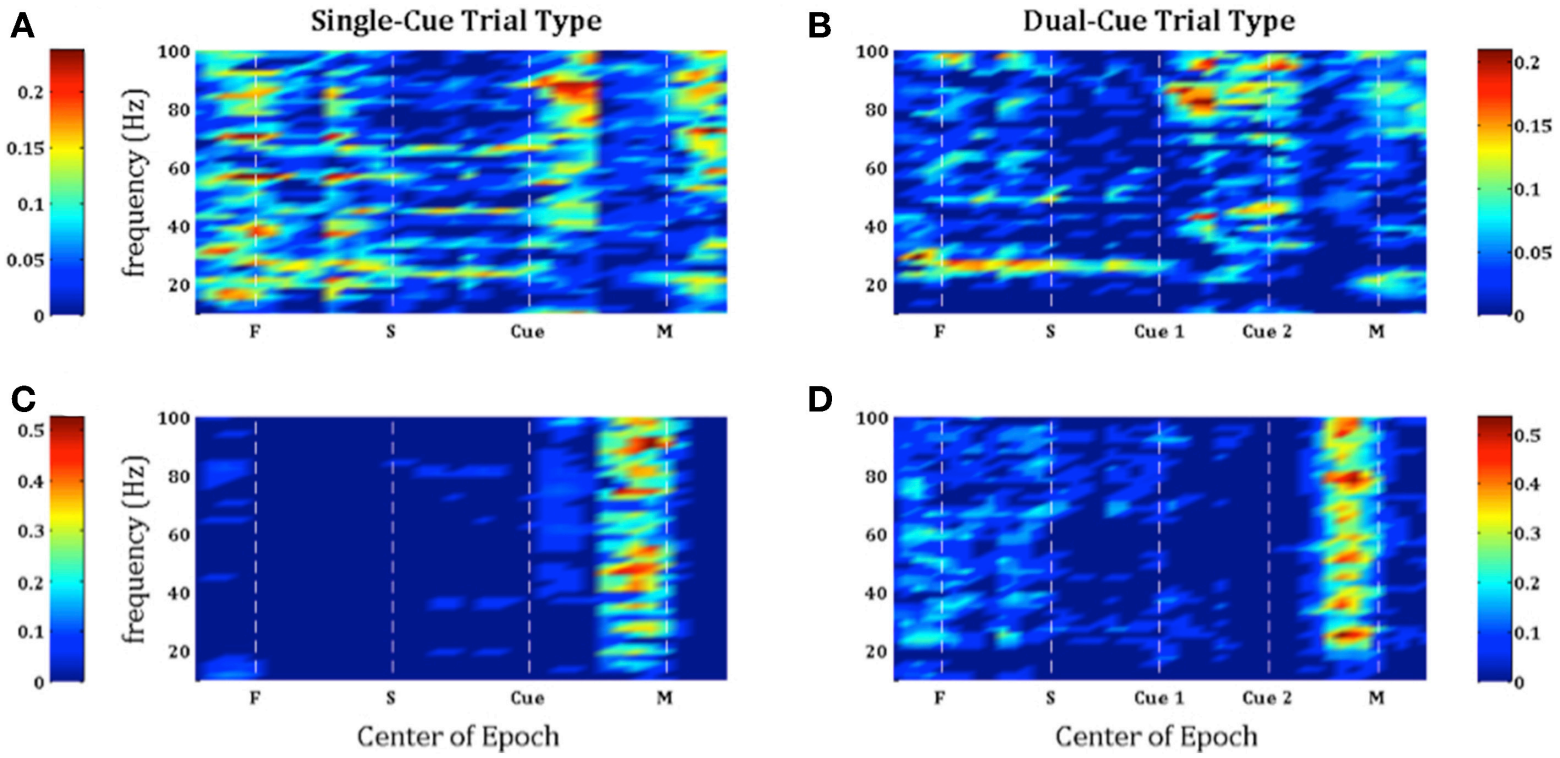
Population-averaged SNR on consecutive windows for directionally tuned neurons **(A,B)** and non directionally tuned neurons **(C,D)** during single-cue tasks **(A,C)** and dual-cue tasks **(B,D)**. For each neuron, only significant frequencies were considered. F, fixation; S, stimulus ON; Cue 1, first cue; Cue 2, second cue; M, movement onset.

**Figure 4:**
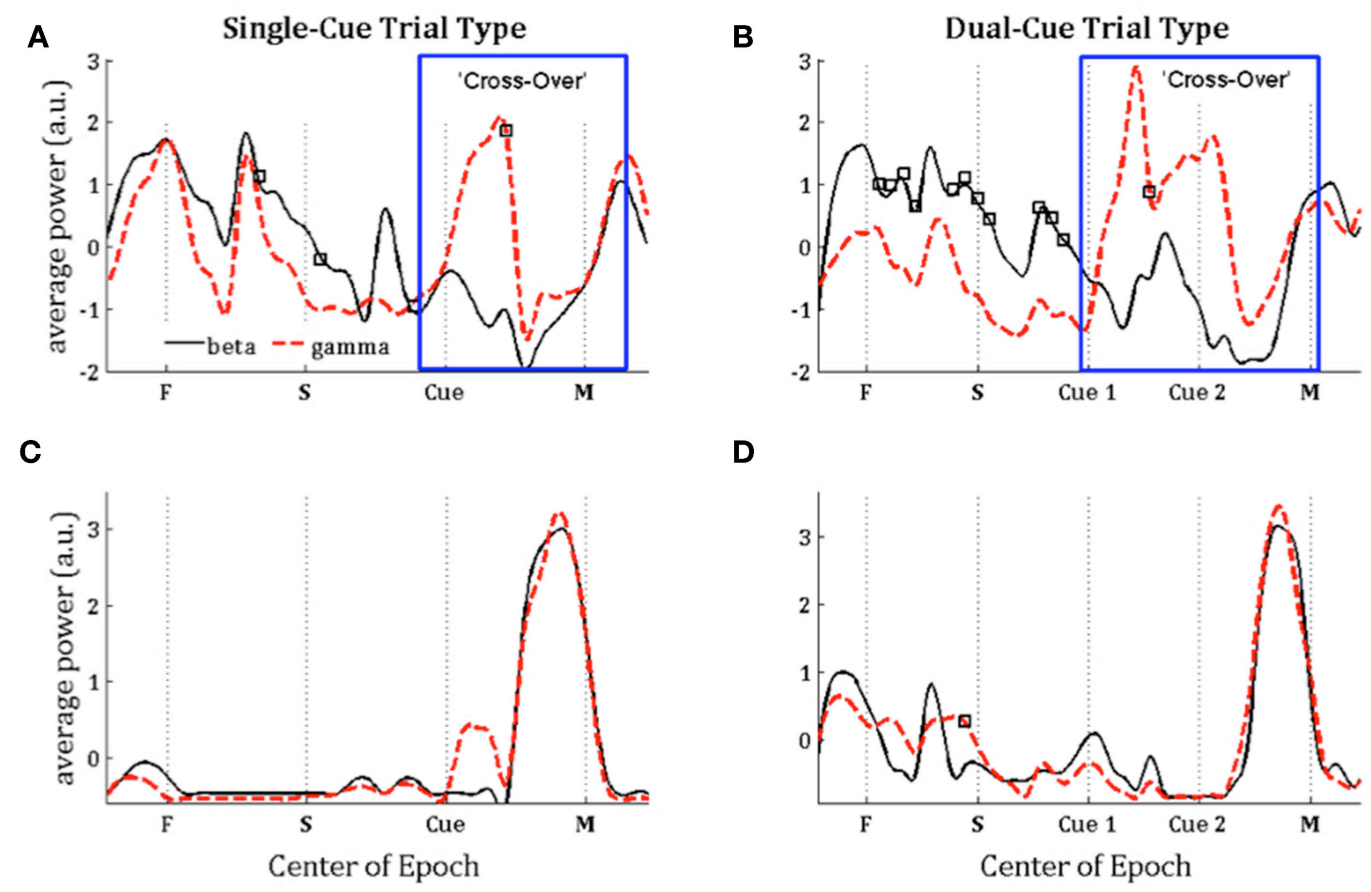
Population-averaged power in the beta (15–30 Hz, black lines) and gamma (35–90 Hz, red lines) frequency band on consecutive windows for directionally tuned neurons **(A,B)** and non-directionally tuned neurons **(C,D)** during single-cue tasks **(A,C)** and dual-cue tasks **(B,D)**. F, fixation; S, stimulus ON; Cue 1, first cue; Cue 2, second cue; M, movement onset. For each window, a black square indicates a significant difference between the power in beta and gamma band in that window (Wilcoxon rank-sum test, *p* < 0.05). Each curve in **(A–D)** is normalized by subtracting the mean value and dividing by the standard deviation.

In Figure 3, we note that the SNR is high throughout the trial in the *single-cue* as well as *dual-cue* directionally tuned neurons. However, before movement we see a sharp drop in SNR across all frequencies. The opposite trend is seen in the non-directionally tuned neurons, with an increase in power across all frequencies. When considering the difference between average power in the gamma and beta frequency bands (Figure 4), we see that the average power in the gamma band is not significantly above that in the beta band until the first cue is observed. After the presence of the first cue in both the single-cue and the dual-cue trial types, we see that the average power in the gamma band is significantly above that in the beta band, till before movement onset. In the case of the dual-cue trials, this “cross-over” effect, i.e., the significantly higher levels of gamma band as compared to the beta band, holds from the onset of the first cue till after the onset of the final cue. Note that no significant differences between the average power in the gamma and beta frequency bands is observed for the non-directionally tuned neurons, see Figures 4C, D. Supplementary Figure 4 shows an example neuron's raster plot during the relevant epochs, as well as the average beta and gamma band power during these epochs. We see that the frequency information is not directly apparent using the rasters alone, but we see the emergence of gamma band and non-emergence or suppression of beta-band power during the relevant epoch.

**Figure 5:**
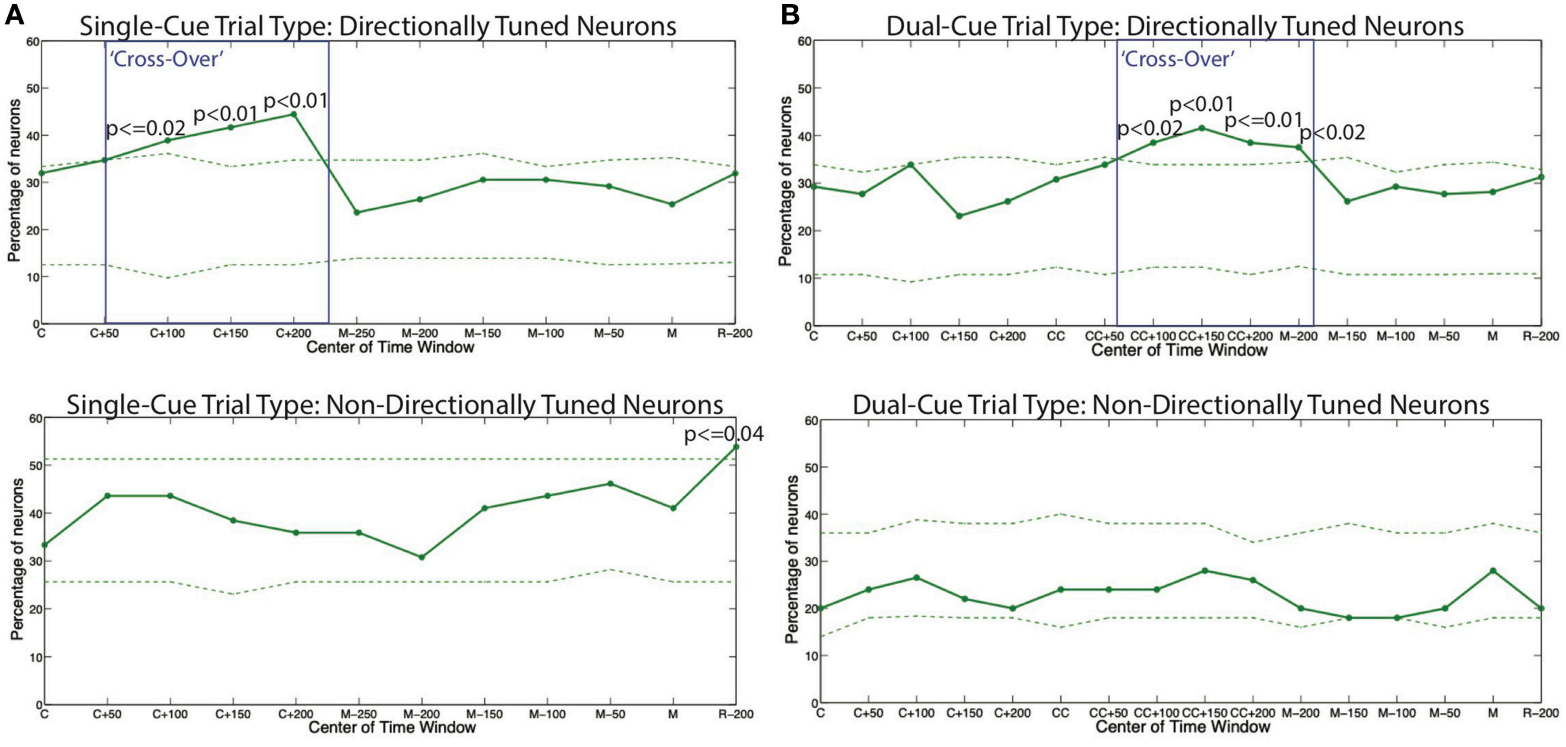
The percentage of all models displaying a higher tendency to oscillate in the gamma frequency band as compared to the beta frequency band for **(A)** the single-cue trial type and **(B)** the dual-cue trial type. The dashed lines show 5 and 95% confidence bounds built by randomly shuffling the inter spike intervals of the original spike trains for each trial of each neuron a total of 100 times. The boxes indicate the areas of cross-over. C, first cue; CC, second cue; M, start of movement; R, administration of reward.

In order to further examine the relationship between the beta and gamma bands after onset of cue, we conducted a series of hypothesis tests to test the exact onset of the cross-over. For each trial-type, we built a separate PPM for each neuron, and calculated the percentage of neurons in each trial type for which the tendency to oscillate in the gamma band is higher than the tendency to oscillate in the beta band, as grouped by directional tuning. This is shown for the *single-cue* and *dual-cue* trial types in Figure 5.

We see that throughout the trials in both trials types, the percentages in the non-directionally tuned neurons do not cross the 5 and 95% bounds computed from the randomized spike trains in a consistent manner. On the other hand, in the directionally tuned neurons, we see that the 95% bounds are crossed by the percentage of neuron model displaying a higher tendency to oscillate in the gamma frequency band as compared to the beta frequency band. This cross-over effect, i.e., the emergence of gamma band and suppression of beta band, holds from the onset of the final cue to 200 ms after the onset of the cue.

Note that this effect is not observed in the directionally tuned neurons after the first cue in the *dual-cue* trials, since these neurons are not tuned in the direction of movement that the first cue suggests. Rather, the cross-over effect in dual-cue trials is seen only after the final cue is presented and lasts until about 200 ms before movement onset.

We also computed the percentage of neuron models displaying a higher tendency to oscillate in the beta frequency band as compared to the gamma frequency band (Supplementary Figure 5). We see that the percentages in both the directionally tuned and the non-directionally tuned neurons do not cross the 5 and 95% bounds computed from the randomized spike trains in a consistent manner.

**Figure 6:**
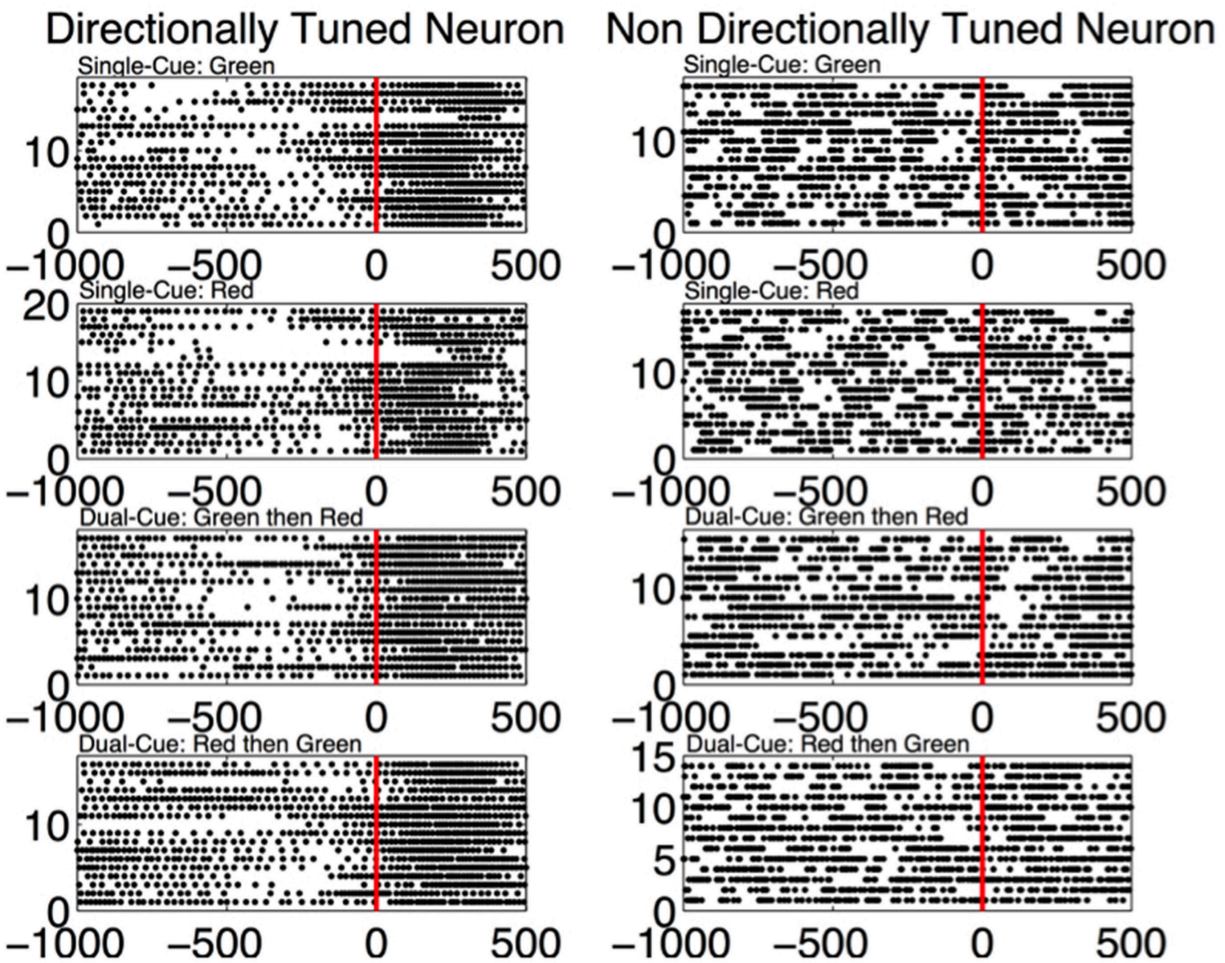
**(Left)** The spiking activity of a directionally tuned neuron 1,000 ms before and 500 ms after the first cue (data aligned at first cue = 0 ms). Only the trials where the monkey is moving in the tuned direction are shown. Each dot corresponds to a spike in the corresponding time bin. **(Right)** The trials from a non-directionally tuned neuron while the monkey is moving in the same direction as the directionally tuned neuron.

In Figure 6, directly after the first cue, we see an emergence of high frequency firing as compared to low frequency firing in the directionally tuned neuron (*p* < 0.05 two-sided Wilcoxon rank sum test of gamma band power as compared to beta band power), supporting the results seen in Figures 4, 5. We do not see these modulations in spiking activity in the non-directionally tuned neuron.

## Discussion

The findings in this study suggest that one mechanism for movement planning in GPi is a specific modulation in the beta-gamma power in the spiking activity of individual neurons. The current theory (Turner and Anderson, 1997; Nambu, 2004) suggests a relationship between movement correlates and the firing rate of neurons, and it is unclear whether the findings in the current study are a causative or a correlative effect of a modulation in the firing rate. Turner & Anderson themselves have performed an extensive study of GPi firing during movements, and found that around 95% of the GPi neurons are tuned to a direction (Turner and Anderson, 1997). Through our directional tuning analysis, we classify 89% of the neurons (71 out of the 80 neurons with cross-validated PPMs) as tuned for at least one movement cue in either of the single-cue or dual-cue trial types. Since we model the complete spiking statistics of a GPi neuron's activity and then test the direction-dependent term for differences in activity, we have a more nuanced measure of directional tuning, and principled comparisons between the two can be performed in a future study.

More specifically, our results show the existence of a “cross-over” effect in the GPi neurons, i.e., a simultaneous increase in gamma band power and decrease in beta band power during movement planning by the task related neurons. It was shown in the results section that the average beta power was significantly higher than the average gamma power before a cue was presented (*p* < 0.05), which then switched to the average gamma band power being significantly higher the average beta band power *after* a cue was presented (*p* < 0.05). Moreover, the existence of beta band oscillatory activity in the task related GPi neurons before the target cue indicates the integral role of this oscillatory activity in achieving accurate directed movements, in contrast with studies involving LFP suggesting that beta band oscillations are inherently pathological (Filion and Tremblay, 1991; Bergman et al., 1994; Raz et al., 2000). In contrast, the non-directionally tuned neurons do not display modulations in the beta or gamma band oscillatory activity until immediately before the onset of movement. During movement, the average power increases concurrently in the beta and gamma bands, with no apparent cross-over effect. Thus, the modulations in oscillatory activity of the task-related or directionally tuned neurons are sharply distinct from those of the non-directionally tuned neurons.

The BG has long been known to participate in movement planning, and the GPi neurons have been shown to consist of directionally tuned and non-directionally tuned neurons (Albin et al., 1989; DeLong, 1990; Mink, 1996; Nambu, 2004; Bromberg-Martin et al., 2010; Shin and Sommer, 2010; Tachibana and Hikosaka, 2012; Howell et al., 2016). Here, we propose that the “directionally-tuned” neurons happen to encode the appropriate course of action, and “non-directionally tuned” neurons represent alternative possible actions. The former are modulated by the ongoing task and the latter are not selectively activated, since those alternative actions are neither appropriate nor tuned for the current context, and are not performed. Moreover, we propose that expression of gamma band activity during motor planning in directionally-tuned neurons amounts to facilitation of the desired or appropriate action given the current state, whereas the expression of beta band activity in non-directionally tuned neurons amounts to suppression of competing but inappropriate or unprofitable actions in the current state and context. This interpretation incorporates the classical ‘center-surround’ hypothesis as well as previously reported beta-band suppression into a coherent theory.

The results of this analysis depend on the use of PPMs to separate the directionally tuned from the non-directionally tuned neurons. PPMs effectively capture the entire spiking activity of each neuron, separating out the relative contribution of history effects and movement direction on the probability that the neuron will spike at any given time, thus making it an effective paradigm to compute directional tuning (Barbieri et al., 2001; Brown et al., 2003; Truccolo et al., 2005, 2008; Sarma et al., 2010; Kahn et al., 2015). Assessing directional tuning using the history-independent covariates of the PPMs is different from simply choosing task-related neurons using firing rates alone, as the PPM separates the contribution of the stimulus and the intrinsic temporal patterns on firing rate (Sarma et al., 2010; Kahn et al., 2015). In fact, traditional means to compute directional tuning rely on first-order statistics of the point process. The PPM parameters instead take into account the probability distributions of the α_*d*_ (not just the mean values), and directional tuning is determined from these distributions.

The modulation in the frequency domain of GPi neurons in healthy animals has not yet been fully investigated in single-neuron studies during movement, to the best of the knowledge of the authors. However, beta and gamma LFP activity and their modulations are long known to have an effect on motor areas, as evidenced by data recorded in various structures during healthy motor control. In one study, it was demonstrated that neural activity in the striatum of awake, behaving macaques is characterized by the presence of widespread synchronous oscillatory activity in the beta band (10–25 Hz) frequency range (Courtemanche et al., 2003). However, as the monkeys performed a visuomotor task in this study, it was found that focal sites could disengage from the beta band oscillations (observed in LFPs) during the time in which neurons at the sites show increased spike activity related to the task. This “pop-out” phenomenon suggests that in the behaving monkey, the temporal structure of ensemble oscillatory activity in the striatum interfaces with a modular spatial organization of task-related activity patterns (Courtemanche et al., 2003). In the human putamen, a similar decrease in beta band power was noted in LFPs with self-paced hand movements (Sochurkova and Rektor, 2003). In addition, numerous EEG studies have demonstrated decrements in beta power and/or increase in gamma power with movements from various regions of the cortex including the primary sensorimotor (Pfurtscheller and Neuper, 1992; Sanes and Donoghue, 1993; Toro et al., 1994; Murthy and Fetz, 1996; Leocani et al., 1997; Donoghue et al., 1998; Alegre et al., 2002; Schoffelen et al., 2005; Donner et al., 2009) and supplementary motor cortex (Leocani et al., 1997; Ohara et al., 2000; Alegre et al., 2002).

The authors propose that the study of oscillatory activity in single neurons in the globus pallidus may provide a novel avenue of analysis for investigating frequency modulations. Although LFP is very useful for studying network oscillations, the recordings integrate signals from multiple neurons (Mitzdorf, 1985; Juergens et al., 1999; Kreiman et al., 2006). It has been suggested in the “funneling” hypothesis by Bergman et al. (1998) that convergence of information takes place from the cortex to the GPi, and a consequent divergence from the GPi back to the cortex, leading to more localized groups of neurons in the GPi that are synchronized in a specific frequency band during a given movement. According to this hypothesis, although LFP recordings from the cortex should show frequency modulation in the relevant bands during movement, especially due to the somatotopic organization of information (Penfield and Rasmussen, 1950; Amirikian and Georgopoulos, 2003), this same effect would not be seen in the GPi neurons since the information at this level should be more locally clustered. Thus, examining the frequency modulation in a group of directionally tuned neurons, that is neurons that modulate their activity in a given movement, is the approach that we implemented in this study.

Our results also concur with studies performed regarding PD conditions in primates. Increased beta band activity in the BG was seen in both the PD human and the monkey treated with the 1-methyl-4-phenyl-1, 2, 3, 6-tetrahydropyridine (MPTP) animal model of Parkinsonism (Filion and Tremblay, 1991; Nini et al., 1995; Levy et al., 2000; Brown et al., 2001; Kuhn et al., 2006; Weinberger et al., 2006). We propose that the reason for impaired motor control in PD may be the inability to perform a cross-over between beta and gamma band oscillations due to pathologically high levels of beta band power present in the basal ganglia. Administering therapies such as Levodopa or deep brain stimulation decreases beta band oscillations in the BG (Priori et al., 2004; Foffani et al., 2005; Wingeier et al., 2006), which may restore the ability to perform a cross-over between beta and gamma band oscillations during the planning of movement, thus restoring healthy motor control.

In conclusion, our results suggest that the parameters of beta-band and gamma-band generation, maintenance and modulation may be pivotal to understanding the mechanism(s) that underlie normal basal ganglia function and may provide insight into the mechanism(s) that underlie pathophysiology of the basal ganglia during PD.

## Ethics Statement

All animal procedures were performed in accordance with the National Institutes of Health guidelines and the Institutional Animal Care and Use Committee approved study protocol.

## Author Contributions

ShS, SrS, EE, and JG contributed to generating ideas, coming up with the hypothesis, and writing the manuscript. JG and EE contributed to experimental procedures. ShS, SP, and SaS contributed to data analysis.

### Conflict of Interest Statement

The authors declare that the research was conducted in the absence of any commercial or financial relationships that could be construed as a potential conflict of interest.
