# Supplementary material for: Modulations in Oscillatory Activity of Globus Pallidus Internus Neurons During a Directed Hand Movement Task—A Primary Mechanism for Motor Planning
Source: Front Syst Neurosci. 2019 Apr 30;13:15. doi: 10.3389/fnsys.2019.00015 (PMC6524693; doi:10.3389/fnsys.2019.00015)
Supplement: Supplementary file 1 [file Data_Sheet_1.pdf]

## *Supplementary Material*

# Modulations in Oscillatory Activity of Globus Pallidus internus Neurons During a Directed Hand Movement Task – A Primary Mechanism for Motor Planning

**Shreya Saxena[1*], Sridevi V. Sarma[2], Shaun R. Patel[3], Sabato Santaniello[4], Emad N. Eskandar[5ϒ], John T. Gale[6ϒ]**

[1] Center for Theoretical Neuroscience, Zuckerman Mind Brain Behavior Institute, Columbia University, New York, NY 10027, USA

[2] Neuromedical Control Systems Laboratory, Institute for Computational Medicine, Department of Biomedical Engineering, Johns Hopkins University, Baltimore, MD 21210, USA

[3] McCance Center for Brain Health, Genetics and Aging Research Unit, Department of Neurology, Massachusetts General Hospital, Harvard Medical School, Boston, Massachusetts, USA

[4] Biomedical Engineering Department and CT Institute for the Brain and Cognitive Sciences, University of Connecticut, Storrs, CT, USA

[5] Leo M. Davidoff Department of Neurological Surgery, Albert Einstein College of Medicine, The Bronx, NY 10461, USA

[6] Department of Neurosurgery, Emory University, Atlanta, GA 30322, USA

[ϒ] **Equally contributing senior authors**

*** Correspondence:** Shreya Saxena, Center for Theoretical Neuroscience, Zuckerman Mind Brain Behavior Institute, Columbia University, New York, NY 10027, USA. **Email:** ss5513@columbia.edu

## 1. Supplementary Figures

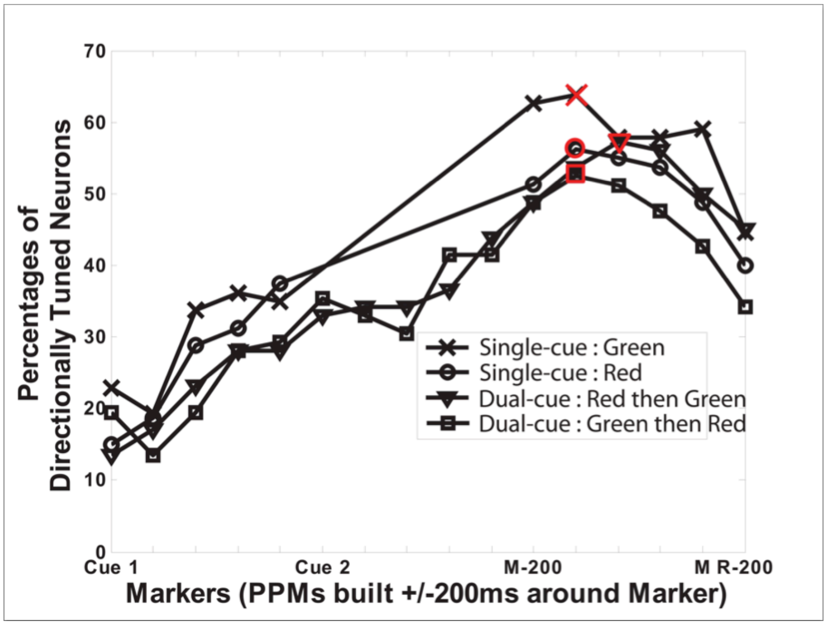


**Supplementary Figure 1.** The percentage of directionally tuned neurons at different epochs for the four movement types. Cue1 corresponds to the first cue seen given to the primate, while Cue2 corresponds to the second cue given (if present). The points displayed in red correspond to the epoch e* containing the highest percentage of directionally tuned neurons.

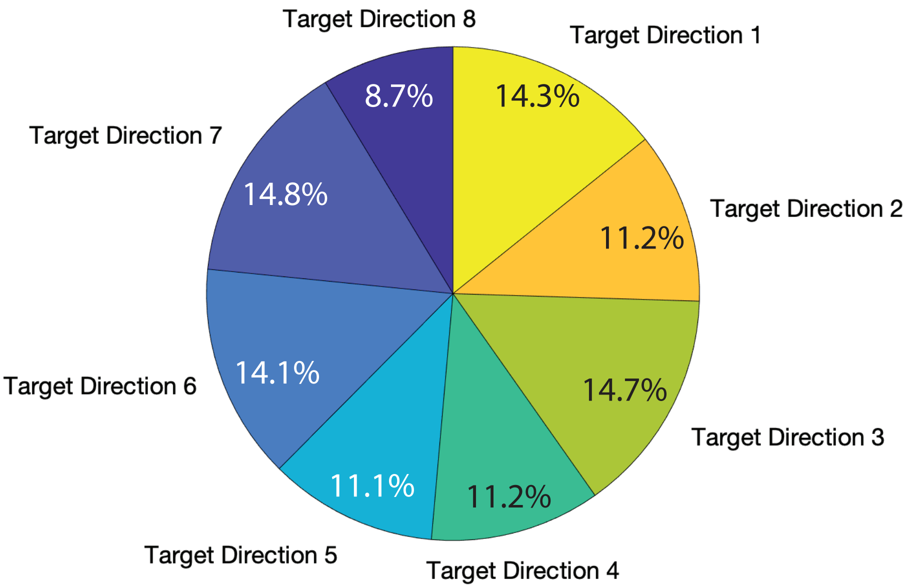


**Supplementary Figure 2.** The percentage of neurons tuned in each movement direction. This consists of the number of neurons that are tuned in each target direction over the total number of tuned neurons. This is an average over all trial types.

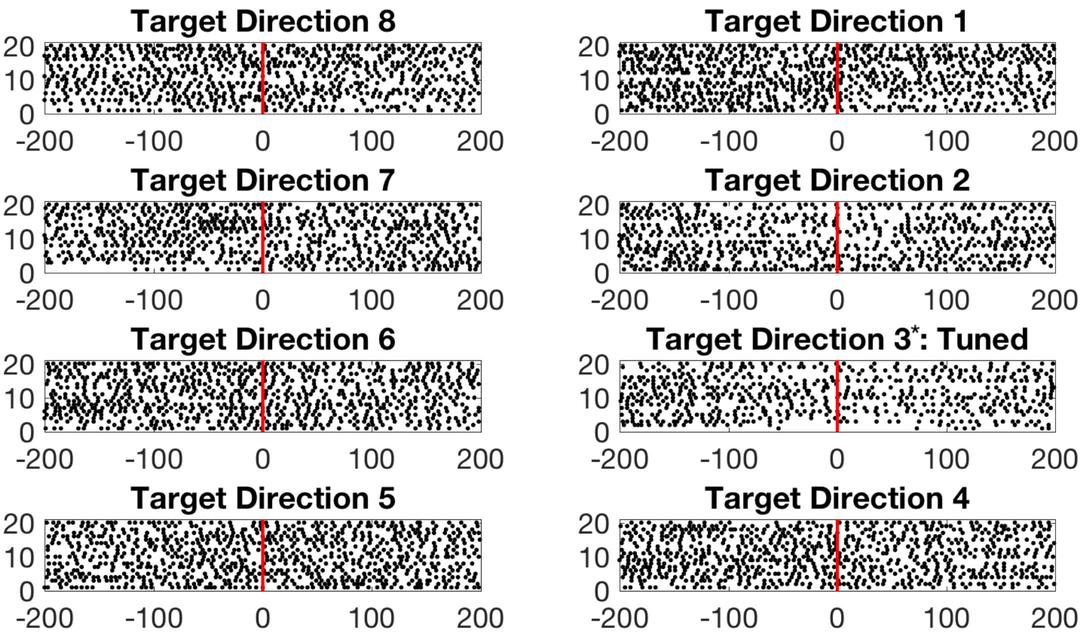


**Supplementary Figure 3.** Spike raster plot of an example directionally tuned neuron around the marker ‘start of movement – 150ms’. This marker is shown as 0 in this plot. This neuron is tuned in target direction 3.

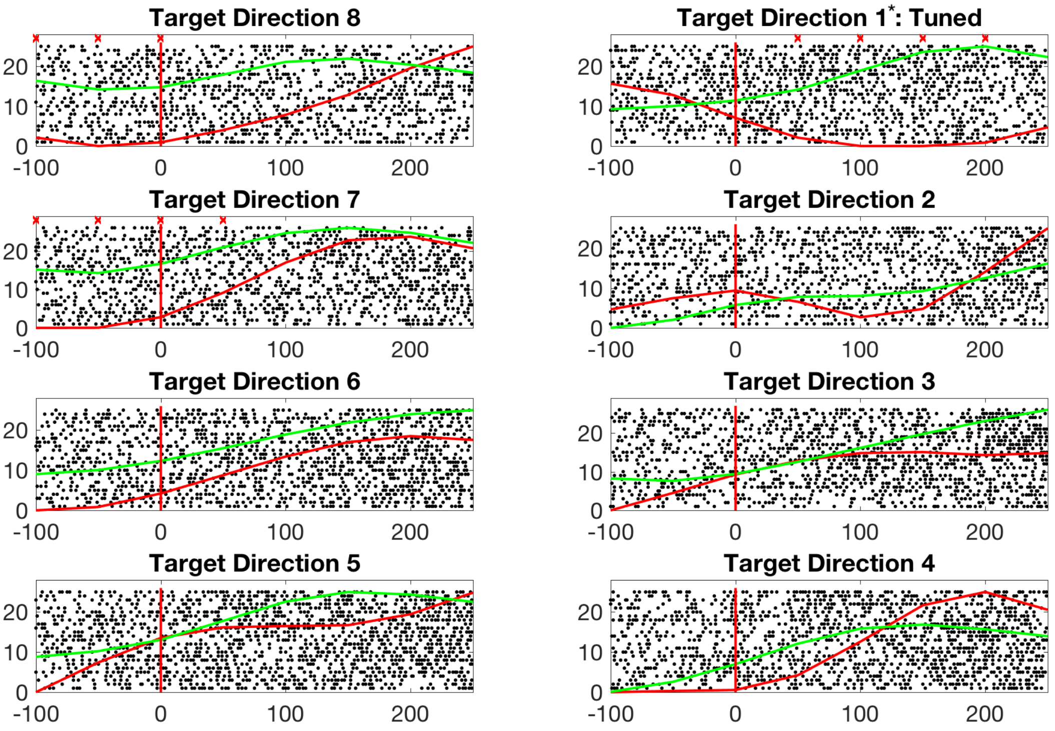


**Supplementary Figure 4.** Spike raster plot of an example directionally tuned neuron around the marker 'cue 1 + 150ms'. This marker is shown as 0 in this plot. This neuron is tuned in target direction 1. The average beta (gamma) power is provided in red (green). The result of a two-sided Wilcoxon rank sum test is shown in red crosses above the rasters, signifying when the median of the power in the beta and gamma bands across trials is significantly different ($p<0.05$).

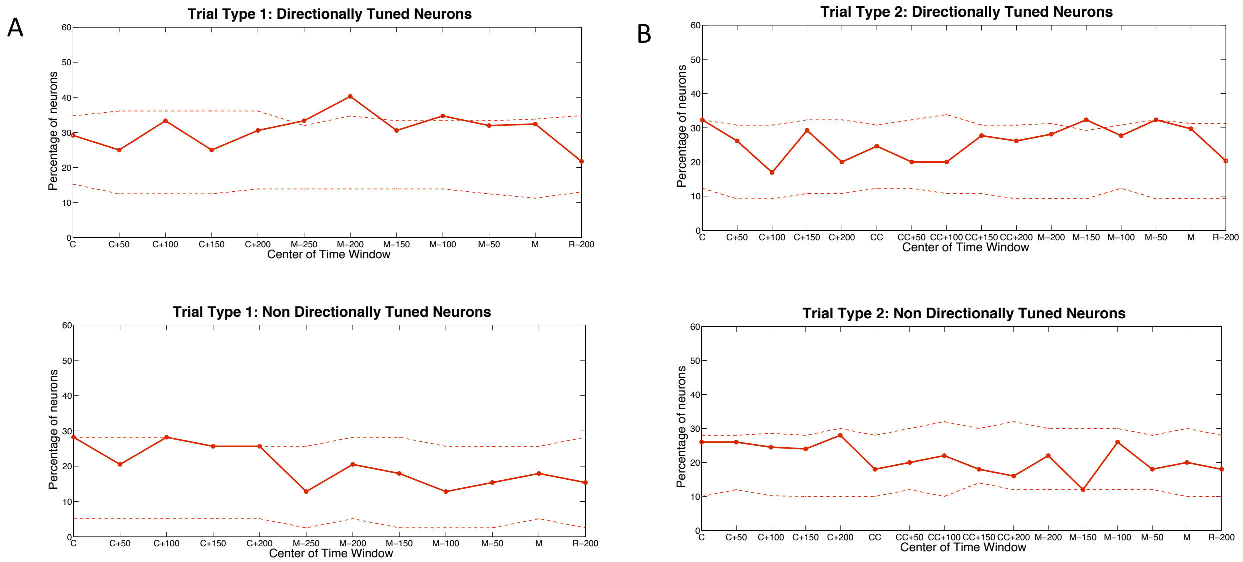


**Supplementary Figure 5.** The percentage of all neurons displaying a higher tendency to oscillate in the beta frequency band as compared to the gamma frequency band for A) the single-cue trial type and B) the double-cue trial type. The dashed lines show 5% and 95% confidence bounds built by randomly shuffling the inter spike intervals of the original spike trains for each trial of each neuron a total of 100 times. C = first cue; CC = second cue; M = start of movement; R = administration of reward.
